# MRA-YOLOv8: A Transmission Line Fault Detection Algorithm Integrating Multi-Scale Feature Fusion

**DOI:** 10.3390/s25247508

**Published:** 2025-12-10

**Authors:** Shuai Hao, Jing Li, Xu Ma

**Affiliations:** 1 College of Electrical and Control Engineering, Xi’an University of Science and Technology, Xi’an 710054, China; haoxust@163.com (S.H.); 18234956351@163.com (J.L.); 2 Shaanxi Aerospace Workers’ University, Xi’an 710018, China

**Keywords:** YOLOv8, multi-scale attention aggregation module, self-attention mechanism

## Abstract

Aiming at the problems of complex background interference and partial occlusion of fault targets during UAV transmission line inspection, this paper proposes an MRA-YOLOv8-based fault detection method for transmission line components. Firstly, the YOLOv8 network is adopted as the baseline framework, and a self-attention mechanism is incorporated into its backbone network to enhance the detection accuracy for occluded targets. Subsequently, a Multi-scale Attention Aggregation module is introduced into the neck network to improve the feature extraction capability for fault targets against complex backgrounds. Furthermore, the bounding box loss function is optimized to mitigate class imbalance issues, thereby boosting the model’s fault detection performance. Finally, the proposed algorithm is validated using inspection data collected over the past three years from an electric power inspection department. Experimental results demonstrate that the proposed method achieves an average detection precision of 92.5% and a recall rate of 90.9%.

## 1. Introduction

In recent years, the continuous expansion of power systems has posed unprecedented challenges to the operation and maintenance of transmission lines, which serve as the “lifelines” of electrical grids. Their extensive coverage and complex operating environments make traditional manual inspection methods—hindered by difficult terrain and harsh conditions—increasingly inefficient, costly, and risky, failing to meet the safety and stability demands of modern power networks [1,2]. Against this backdrop, UAV-based inspection technology represents a significant advancement in the field. Furthermore, by integrating emerging technologies such as artificial intelligence and big data, UAVs can achieve automated defect detection and intelligent fault diagnosis of transmission lines, which in turn significantly improves the overall intelligence and efficiency of grid operation and maintenance [3].

UAV-powered inspection technologies have become a vital component of modern transmission network maintenance strategies [4]. Vision-based fault detection methods for transmission lines can be broadly categorized into two groups: traditional machine learning approaches [5,6] and deep learning-based methods [7,8].

Object detection algorithms based on convolutional neural networks can be broadly categorized into two groups: anchor-based and anchor-free methods. Anchor-based approaches can be further subdivided into one-stage and two-stage detectors [9]. Two-stage algorithms divide the detection process into two phases: generating region proposals containing potential objects, followed by object classification and bounding box regression. Representative algorithms include R-CNN [10], Fast R-CNN [11], and Faster R-CNN [12]. However, the sequential nature of proposal generation and subsequent refinement necessitates multi-step processing, resulting in slower training and inference speeds. This increased computational time makes them less suitable for applications requiring high real-time performance.

In contrast, one-stage algorithms directly predict both object categories and locations in a single forward pass, eliminating the need for a separate region proposal stage. This significantly reduces computational complexity and improves detection speed. Classical examples include SSD (Single Shot MultiBox Detector) [13] and the YOLO series (You Only Look Once) [14,15,16,17,18]. For instance, Zhang et al. proposed an improved SSD algorithm that integrates multi-level features via a channel attention mechanism and dilated convolutions, thereby expanding the receptive field of low-level features [19]. Xu et al. applied YOLOv5 for the automatic annotation of insulator images, where the network was first fine-tuned on a small set of manually labeled examples and then used to automatically label larger datasets [20]. Ma et al. incorporated an SPPCSPC module into the backbone network to aggregate multi-scale features within a spatial pyramid, enhancing the model’s capability to detect objects at various scales [21]. Despite numerous advances in fault detection for transmission lines, most existing studies focus on improving single-stage detection accuracy or lightweight model optimization [22,23,24]. However, challenges such as partial occlusion, small fault scale, and complex background interference remain unresolved. Recent studies [25,26,27] attempted to enhance YOLO-based architectures but often overlook feature correlation modeling and multi-scale aggregation, limiting their ability to handle fine-grained or occluded faults. To address these gaps, this study proposes MRA-YOLOv8, which integrates self-attention mechanisms and multi-scale attention aggregation, coupled with an adaptive loss design, to achieve robust fault detection under challenging UAV inspection scenarios. The main contributions of this work are as follows:

(1) To address the issue of weakened features for small targets against complex backgrounds, a Multi-Scale Attention Aggregation (MSAA) module is designed. In the spatial refinement path, multi-scale fusion helps retain fine-grained features of small objects, while the channel aggregation path enhances the model’s focus on channel information that is most relevant to the target. Together, these two mechanisms improve background suppression and effectively reduce interference from cluttered environments.
(2) To address the performance degradation caused by partial object occlusion, a C2f-Restormer module is introduced. This module captures inter-feature relationships across different subspaces, computes attention weights to assess element importance for adaptive feature aggregation, and models long-range dependencies. Consequently, it significantly enhances the overall feature representation capacity.
(3) This study proposes ATFL (Adaptive Task-focused Focal Loss), a loss function that adaptively adjusts training weights based on task-specific requirements, thereby improving model performance and convergence. It is intended to alleviate class imbalance issues and increase the model’s focus on hard-to-classify examples, thereby improving fault detection performance.

## 2. Principle of YOLOv8 Detection Algorithm

The advancements in the field of deep learning have facilitated the continuous development of the YOLO series, aiming to achieve higher detection accuracy and more efficient feature representation. Building on the foundation of YOLOv5, YOLOv8 [28] further enhances the network architecture by incorporating the SPP (Spatial Pyramid Pooling) module and the PAN (Path Aggregation Network) module, thereby improving feature extraction, multi-scale representation, and overall detection performance.

(1) Input: Input images of varying sizes are initially resized to a uniform resolution of 640 × 640 to meet the model’s input requirements. Data diversity is enhanced through augmentation techniques such as random cropping, rotation, and flipping, thereby improving dataset quality. Predefined anchor boxes are utilized to generate candidate regions, preparing the data for subsequent feature extraction and object detection.
(2) Backbone: Serving as the core component of YOLOv8, the backbone network extracts multi-level features from the input images. By incorporating a Cross-Stage Partial (CSP) structure, it effectively reduces computational overhead while maintaining strong feature representation capability. The use of depthwise separable convolution further decreases the number of parameters and computational complexity. Hierarchical convolutional operations capture features at different scales, enabling robust detection of multi-scale objects.
(3) Neck: YOLOv8 employs a Path Aggregation Network (PANet) as its neck module. This architecture facilitates bidirectional (top-down and bottom-up) multi-scale feature fusion, enriching feature expressiveness. By integrating contextual information and combining features from different levels, the model gains the ability to detect objects across a wide range of scales, particularly enhancing its performance on small targets.
(4) Head: The detection head constitutes the final component of YOLOv8, responsible for generating the final detection results. Its primary functions include classification and regression, multi-task learning, and non-maximum suppression (NMS). Classification and regression involve predicting object categories and bounding box coordinates for each candidate region. Through multi-task training, end-to-end object detection is achieved. Finally, the NMS algorithm is applied to eliminate redundant detection boxes and retain the most probable predictions.

## 3. MRA-YOLOv8 Detection Network

**Figure 1:**
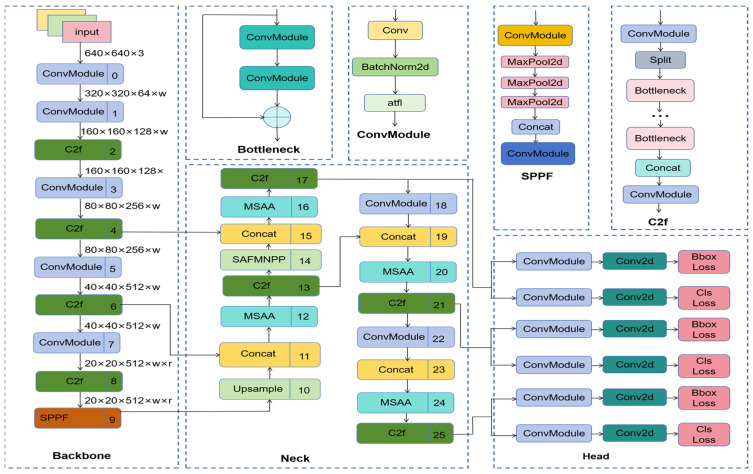
MRA-YOLOv8 transmission line fault target detection network framework.

The MRA-YOLOv8 transmission line fault target detection network framework proposed in this paper is shown in Figure 1.

### 3.1. Backbone Network Reconfiguration

During the detection of faulty components in transmission lines, some objects are notably small and exhibit high similarity to their surrounding environment, making accurate differentiation challenging. To address this issue, we integrate the C2f and Restormer modules to form a unified C2f-Restormer block. Additionally, a Multi-Scale Attention Aggregation (MSAA) module is incorporated to mitigate the adverse impact of complex backgrounds on detection accuracy. Furthermore, to enhance the model’s focus on defect categories, especially those that are challenging to detect under difficult conditions, an Adaptive Task-focused Focal Loss (ATFL) is introduced, thereby improving overall recognition performance.

#### 3.1.1. Multi-Scale Attention Aggregation Module (MSAA)

In complex environments, the detection of small targets is often hindered by weakened feature representation and significant background interference, which collectively degrade detection accuracy. The MSAA module [29] tackles these issues through multi-scale fusion and channel-wise aggregation pathways, effectively improving the detection efficacy of small objects. By leveraging multi-dimensional fusion techniques, the module captures feature information across different scales, enabling precise identification of small targets under varying background conditions. Simultaneously, the integration of information from different feature channels enhances the expressive capacity of small target features, thereby increasing robustness in cluttered scenes. The MSAA module not only strengthens the recognition capability for small targets but also significantly suppresses background interference in complex environments, offering an effective solution for small object detection.

**Figure 2:**
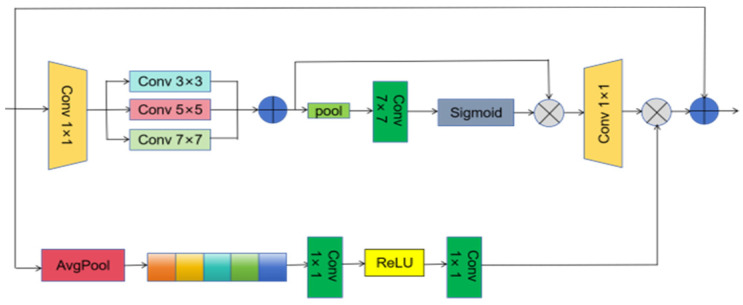
Structure of the Multi-dimensional Attention Aggregation (MSAA) module.

The features extracted from the backbone network undergo a refinement process through dual-path operations on both spatial and channel dimensions, enhancing the informational content in each domain and ultimately improving the quality of the output feature map. In the spatial refinement path, the number of channels is first reduced to C_2_ via a 1 × 1 convolution. Multi-scale features are then aggregated by concatenating outputs from convolutional layers with varying receptive fields (3 × 3, 5 × 5, 7 × 7). These features are subsequently combined using both average and max pooling operations. A spatial attention map is generated by applying a sigmoid activation following a 7 × 7 convolution, which is then multiplied element-wise with the original features to emphasize salient regions. Simultaneously, in the channel aggregation path, global average pooling reduces the spatial dimensions to 1 × 1, producing a compact channel-wise descriptor. This descriptor is then processed by a 1 × 1 convolution followed by a ReLU activation to generate channel attention weights. The resulting channel attention mechanism is fused with the spatially refined features through multiplicative integration. By incorporating the MSAA module, the model demonstrates improved recognition accuracy for small targets and enhanced overall detection efficiency, see Figure 2.

#### 3.1.2. Self-Attention Mechanism Restormer

Leveraging the self-attention mechanism [30], the module establishes inter-dependencies among spatial features, computes attention weights to integrate information, and captures long-range contextual relationships. The incorporation of local encoding techniques provides positional information to the model, facilitating the recovery of structural details within the image.

**Figure 3:**
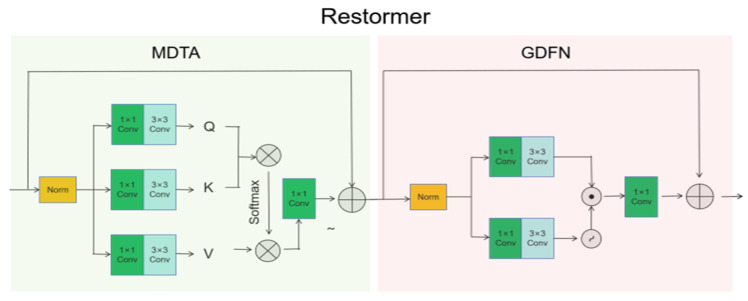
Restormer module with self-attention mechanism.

The module comprises two principal components: the Multi-scale Dense Transformer Attention (MDTA) and the Gated Dual-linear Feedforward Network (GDFN), which collectively form a hierarchical feature extraction architecture. Within this framework, internal features are processed through multiple parallel pathways. Each pathway independently computes feature representations, which are subsequently merged and transformed to enhance representational capacity. The GDFN further refines the output features from the MDTA through a gating mechanism and bilinear transformation. This enables controlled feature flow and adaptive fusion, allowing selective enhancement or suppression of different feature responses to optimize the final output, see Figure 3.

#### 3.1.3. Improved Bounding Box Loss Function

The Adaptive Threshold Focal Loss (ATFL) [31] is a loss function designed to dynamically adjust loss weights during training. Its primary objective is to reduce the model’s reliance on easily classifiable examples while increasing its focus on hard-to-classify instances, thereby improving detection performance in class-imbalanced scenarios.

ATFL automatically modulates loss weights based on each example’s distinctive characteristics and the model’s current predictions. By leveraging prior knowledge and self-learning capabilities from the data, it determines optimal loss weights through individualized training for each sample. The model dynamically adjusts a classification threshold based on the discrepancy between predicted outputs and ground-truth labels. This mechanism allows it to allocate greater attention to challenging examples during the training phase, ultimately enhancing the model’s robustness and fault detection capability. When Pt≤0.5, the formula is as follows:

(1) $$\mathrm{ATFL}=-(\lambda -pt{)}^{-\ln(pt)}\log(pt)$$

When pt>0.5, the formula is as follows:

(2) $$\mathrm{ATFL}=-(1-pt{)}^{-\ln(\hat{p}c)}\log(pt)$$

Among these, pt indicates the current average predicted probability value. $\hat{p}c$ indicates the forecast value for the next round.

## 4. Results and Analysis

The hardware configuration used in the experiments is listed in Table 1.

### 4.1. Dataset and Model Training

**Figure 4:**
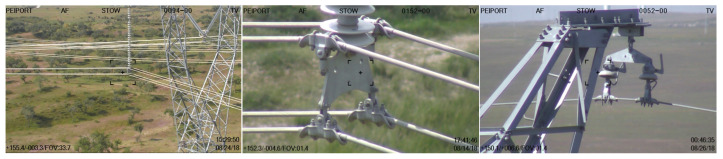
The unmanned aircraft collected examples of faulty components at different heights.

The dataset used in this study was collected from a power station over the past three years, covering various weather conditions, seasons, different time periods, and lighting angles. These images were captured by drones flying at different heights and from different perspectives, and multiple images at different heights were obtained by adjusting the camera focal length to present targets of different scales and perspectives. A total of 2001 images were used for training, 482 for testing, and the training method of this model was from scratch. The following picture shows some examples, see Figure 4.

The types of the database and the number of examples in it are shown in Table 2.

**Figure 5:**
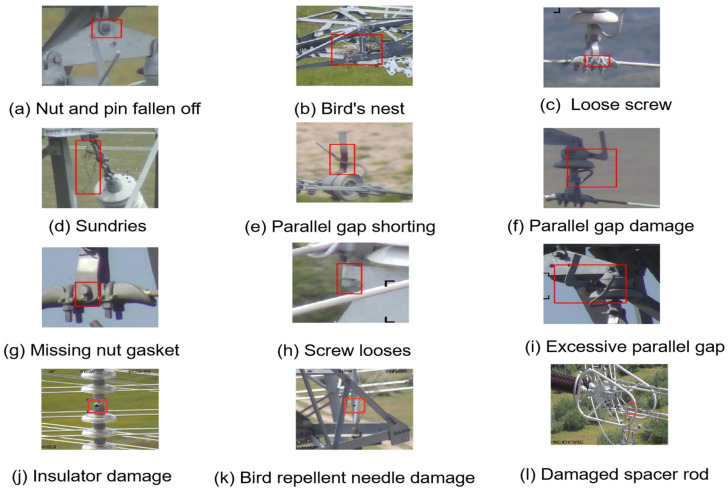
Example images of different fault types in the transmission line dataset.

Figure 5 illustrates representative examples of the 12 fault categories listed in Table 2.

During model training, the batch size was set to 16, the number of epochs to 200, and the initial learning rate to 0.001.

### 4.2. Test Results and Analysis

To comprehensively assess the performance of the proposed algorithm, recall and precision are adopted as evaluation metrics. The corresponding formulas for these parameters are given below [32,33]:

(3) $$mAP=\sum_{i=1}^{N}APi/N$$

(4) $$\mathrm{Recall}=\frac{N_{\mathrm{TP}}}{N_{\mathrm{TP}}+N_{\mathrm{FN}}}$$

(5) $$\mathrm{Precision}=\frac{N_{\mathrm{TP}}}{N_{\mathrm{TP}}+N_{\mathrm{FP}}}$$

The above formula, $N_{\mathrm{TP}}$, $N_{\mathrm{FP}}$, $N_{\mathrm{FN}}$ Correct detections, false positives, and false negatives, respectively. The line integral of AP with respect to P-R; N denotes the number of detection categories, i∈(0,N).

**Figure 6:**
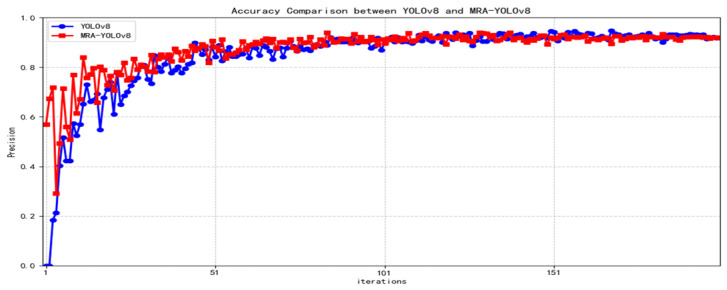
Accuracy contrast curve.

**Figure 7:**
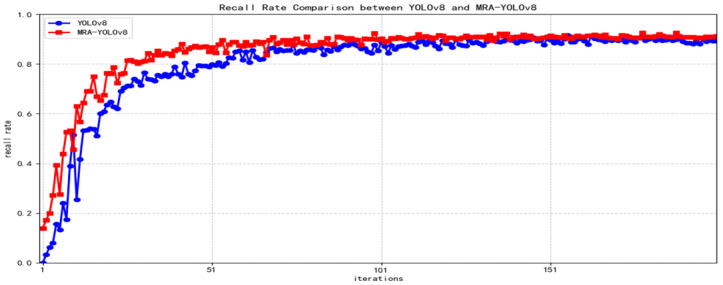
Curve comparing recall rates.

**Figure 8:**
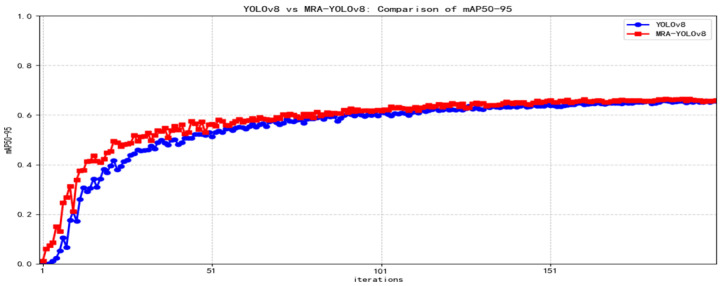
mAP50-95 contrast curve.

The proposed algorithm’s accuracy, recall, and mAP50-95 curves are compared with the original YOLOv8 as shown in Figure 6, Figure 7 and Figure 8 below.

The proposed algorithm incorporates a multi-scale attention aggregation module, a self-attention mechanism module, and an improved bounding box loss function. To better evaluate the effectiveness of each component module, ablation experiments were conducted, and the results are presented in Table 3.

All performance metrics include precision rate, recall rate, mAP50 and mAP50–95. The precision rate measures the proportion of correctly predicted positive samples among all positive samples predicted, while the recall rate measures the proportion of positive samples that are correctly detected among all actual positive samples. mAP50 represents the average mean average precision at an IoU threshold of 0.5, and mAP50–95 is the average value of AP scores calculated over the range of IoU thresholds from 0.5 to 0.95, providing a basis for comprehensive evaluation under different levels of detection strictness. For all these metrics, the higher the value, the better the performance. Integrating the MSAA and C2f-Restormer modules enhances the feature extraction capability, especially for small-scale targets and complex backgrounds, thereby achieving improvements in precision rate, recall rate and mAP metrics.

As can be observed from Table 3, the detection precision of the baseline YOLOv8 network for fault targets is 0.902. Given the diversity of transmission line fault types and detection scenarios, specific enhancements were designed to address challenges such as complex backgrounds, partial occlusions, and small-scale targets: the C2f-Restormer module, the MSAA module, and an improved bounding box loss function. Incorporating only one or two modules may bias feature extraction toward certain fault characteristics, overlooking other critical features and slightly reducing performance. Furthermore, for fault samples that are difficult to identify due to challenging conditions such as varying illumination or occlusions, the ATFL function enables the model to focus more effectively on learning discriminative features from these hard examples, thereby improving detection accuracy.

**Figure 9:**
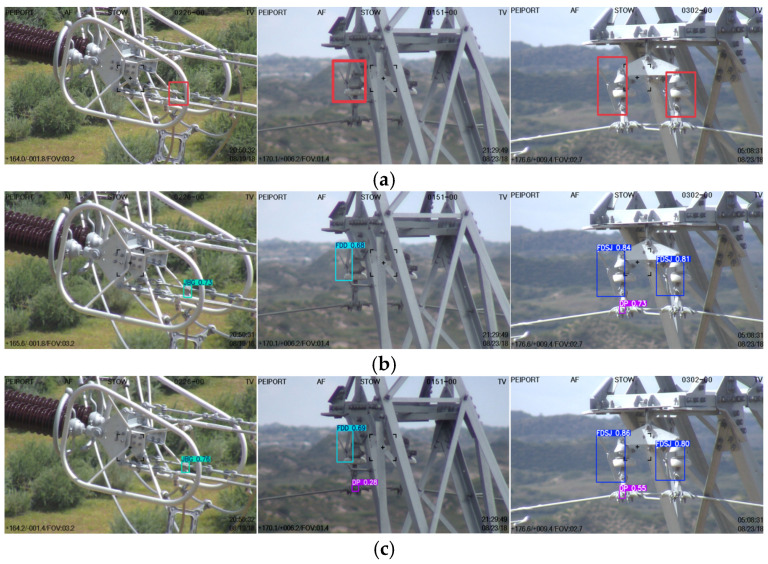

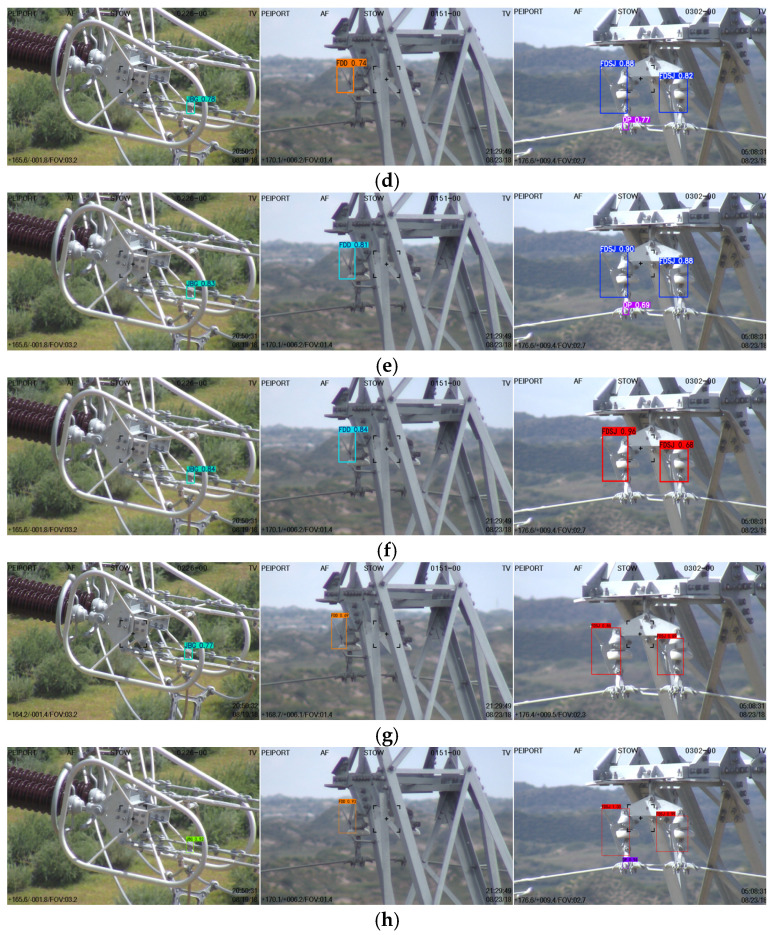
Comparison of test results. Ground Truth. (**a**) Ground Truth, (**b**) SSD, (**c**) Faster RCNN, (**d**) Center Net, (**e**) YOLOv5, (**f**) YOLOv8, (**g**) YOLOv8n, (**h**) MRA−YOLOv8.

To further validate the advantages of the proposed MRA-YOLOv8 algorithm, evaluations were conducted under three distinct challenging scenarios: partially occluded fault targets, small-scale targets, and targets in complex backgrounds. The detection results are illustrated in Figure 9, with detailed outcomes presented for the three experimental groups below.

In the first group experiment, the faulty component was partially occluded. Comparative results from the first experimental group indicate that both YOLOv5 and SSD demonstrate relatively low accuracy in detecting partially obscured objects. The proposed algorithm addresses this limitation by employing a self-attention mechanism, which captures dependencies among spatial features, computes attention weights to evaluate the importance of each element during information integration, and models long-range contextual relationships. As a result, the method exhibits increased robustness and accuracy when detecting fault targets subject to partial occlusion.

In the second group experiment, the faulty component was a small-scale one. In UAV-based inspection, images are often affected by various disturbances, causing small targets to appear blurred and difficult to recognize. The proposed algorithm incorporates the MSAA module to preserve discriminative features of small-scale objects. This module performs multi-dimensional fusion through spatial refinement and enhances the focus on target-relevant characteristics via channel-wise aggregation. Consequently, detection precision for small-scale anomalies is significantly improved.

In the third group experiment, the target was in a complex background. In complex backgrounds, imprecise feature matching often leads to missed detections or inaccurate localization. Experimental results show that while conventional detectors such as YOLOv5, YOLOv8, SSD, and Faster R-CNN are capable of identifying faults in such environments, their detection accuracy remains limited. The proposed method improves feature fusion efficiency through a multi-dimensional combination of spatial and channel-wise mechanisms, thereby enhancing robustness to complex scenes, suppressing background interference, and increasing fault detection accuracy under challenging conditions.

To objectively evaluate the performance of the proposed method, comparative experiments were carried out against several detection networks, including YOLOv5, YOLOv8, CenterNet, SSD, and Faster R-CNN. The corresponding results are summarized in Table 4.

As shown in Table 4, the proposed algorithm achieves a higher mAP50 than the other algorithms, fully validating its superiority. Compared with the baseline YOLOv8 model, MRA-YOLOv8 achieves an inference time of 13.7 ms per image, which is approximately 22% slower than YOLOv8 (11.2 ms) due to the added attention modules.

**Figure 10:**
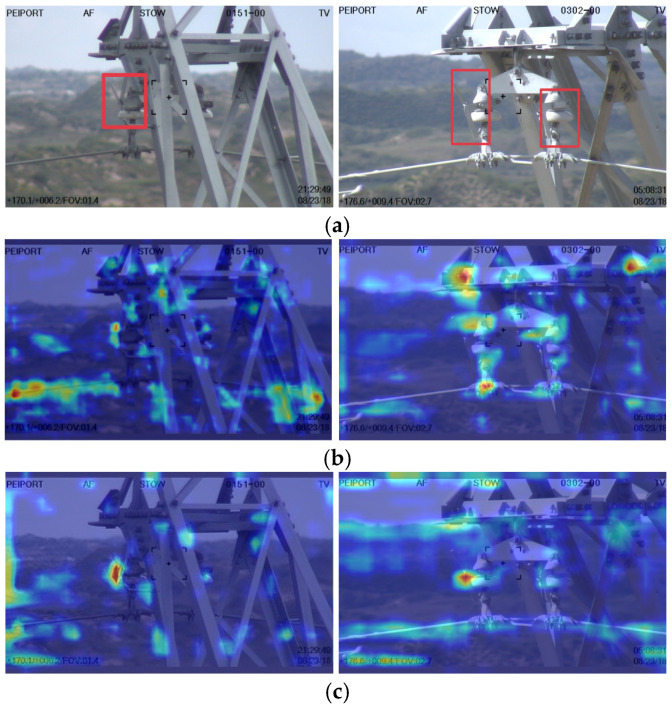

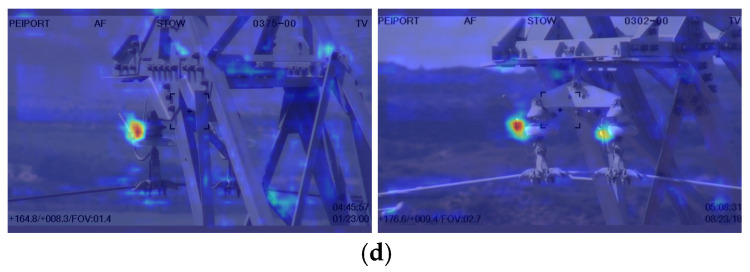
Visualization of attention heatmaps for occluded fault targets. (**a**) Original image, (**b**) CBAM, (**c**) SE, (**d**) MSAA.

To further illustrate the enhanced feature focus of MRA-YOLOv8, Figure 10 visualizes attention heatmaps generated from the C2f-Restormer and MSAA modules. As shown, the proposed network concentrates more effectively on occluded or partially visible fault regions compared with the baseline YOLOv8, confirming that the attention mechanisms facilitate robust contextual reasoning.

## 5. Conclusions

This study presents MRA-YOLOv8, a tailored YOLOv8-based algorithm for fault detection in UAV-assisted transmission line inspection. The proposed approach integrates multiple specialized modules to enhance feature representation, improve detection robustness, and optimize overall performance. The principal contributions and findings are summarized as follows:

(1) The C2f-Restormer self-attention module enhances global feature extraction and captures long-range dependencies in transmission line images, thereby improving the detection of small and partially occluded fault instances. This enhancement is accompanied by a moderate increase in GPU memory usage, which is considered acceptable given the current focus on accuracy.
(2) The MSAA module facilitates multi-scale feature fusion by emphasizing informative features across different resolutions, thereby enhancing the model’s ability to detect faults of varying sizes under complex environmental conditions.
(3) The ATFL dynamically adjusts training weights according to task-specific requirements, mitigating class imbalance and improving precision and recall for challenging fault instances.

Experimental results indicate that MRA-YOLOv8 achieves a precision of 92.5%, recall of 90.9%, and mAP50 of 0.93, surpassing state-of-the-art detectors such as YOLOv8 and Faster R-CNN. The model demonstrates an effective balance between detection accuracy and real-time performance, making it highly suitable for practical UAV inspection applications. Despite the improved detection robustness, the inclusion of the C2f-Restormer module increases computational cost. Future work will focus on incorporating lightweight attention mechanisms, cross-domain adaptation, and multi-sensor fusion to further enhance robustness, deployability, and real-time applicability for operational UAV inspection systems.

## Figures and Tables

**Table 1:** The test environment and related parameters.

Configuration	Version Information
CPU	i5-13490F
GPU	RTX 4060ti
Operating System	Windows 11
Deep learning framework	Pytorch2.0.1 CUDA 11.8

**Table 2:** Type and number of datasets.

Fault Type	Custom Name	Number of Faults
Damage to parallel gap	FDSJ	152
Short circuit in parallel gaps	FDD	174
Excessive parallel gaps	FDG	230
Spacer rod dislodged	JBG	284
Pin dislodged	XT	252
Screw dislodged	LST	267
Insulator dislodged	JYT	162
Power lines obstructed by debris	ZW	178
Power lines obstructed by bird nests	NC	145
Nut missing washer	DP	263
Nut loose	LSS	260
Bird deterrent spikes damaged	NS	116

**Table 3:** Ablation test results.

C2f-Restormer	MSAA	ATFL	Precision	Recall	mAP50	mAP50-95
-	-	-	0.902	0.889	0.910	0.627
√	-	-	0.918	0.932	0.935	0.677
-	√	-	0.941	0.881	0.923	0.641
-	-	√	0.911	0.887	0.924	0.653
√	√	-	0.933	0.902	0.920	0.681
√	-	√	0.924	0.923	0.937	0.670
-	√	√	0.934	0.914	0.935	0.671
√	√	√	0.925	0.909	0.93	0.917

“√” indicates that the module is included, while “-” indicates that the module is not included.

**Table 4:** Training results of the different algorithms.

Algorithm	Precision	Recall	mAP50	mAP50-95	Time (ms)
YOLOv5	0.903	0.874	0.907	0.638	14.5
Faster RCNN	0.88	0.843	0.78	0.552	28.7
SSD	0.874	0.825	0.756	0.54	13.9
YOLOv8	0.918	0.892	0.92	0.655	11.2
Center Net	0.895	0.87	0.81	0.575	16.1
YOLOv8n	0.866	0.81	0.746	0.52	10.1
MRA-YOLOv8	0.925	0.909	0.93	0.917	13.7

## Data Availability

Restrictions apply to the availability of these data. Data were obtained from power grid bureau and are available from the authors with the permission of power grid bureau.
